# Impact of urinary incontinence on health-related quality of life, daily activities, and healthcare resource utilization in patients with neurogenic detrusor overactivity

**DOI:** 10.1186/1471-2377-14-74

**Published:** 2014-04-04

**Authors:** Derek H Tang, Danielle Colayco, James Piercy, Vaishali Patel, Denise Globe, Michael B Chancellor

**Affiliations:** 1 The University of Arizona College of Pharmacy, 1295 N. Martin, PO Box 210202, Tucson, AZ 85721, USA; 2 Department of Global Health Outcomes Strategy and Research, Allergan Inc, 2525 Dupont Drive, Irvine, CA 92612, USA; 3 Adelphi Real World, Adelphi Mill, Grimshaw Lane, Bollington SK10 5JB, UK; 4 Oakland University William Beaumont School of Medicine, Neurourology Program, Beaumont Hospital, 3535 West 13 Mile Road, Royal Oak, MI 48073, USA

**Keywords:** Neurogenic detrusor overactivity, Incontinence, Burden of illness, Quality of life, Productivity

## Abstract

**Background:**

Neurogenic detrusor overactivity (NDO) leads to impaired health-related quality of life (HRQoL), productivity, and greater healthcare resource burden. The humanistic and economic burden may be more apparent in NDO patients with urinary incontinence (UI). The objective of this study was to compare the HRQoL, productivity, and health resource use (HRU) between continent and incontinent NDO patients.

**Methods:**

A retrospective database analysis was conducted using the Adelphi Overactive Bladder (OAB)/UI Disease Specific Programme, a multi-national, cross-sectional survey reported from both patients’ and physicians’ perspectives. The population for this analysis included NDO patients with or without UI. General and disease-specific HRQoL were assessed using the EuroQoL-5D (EQ-5D), Incontinence Quality of Life questionnaire (I-QOL), and the Overactive Bladder Questionnaire (OAB-q). Productivity and daily activity impairment were measured using the Work Productivity and Activity Impairment (WPAI) questionnaire. HRU indicators included OAB-related surgery, OAB-related hospitalizations, incontinence pad usage, switching anticholinergics used for OAB due to inadequate response or adverse effects, and OAB-related physician visits. Bivariate analyses, multivariate ordinary least squares (OLS) regression analyses and published minimal clinically important differences (MCID) were used to assess relationships between incontinent status and the aforementioned outcome measures.

**Results:**

A total of 324 NDO patients with or without urinary incontinence were included, averaging 54 years of age (SD 16), of whom 43.8 percent were male. Bivariate analyses detected no significant relationship between incontinent status and HRU variables. Regression analyses revealed that incontinent patients had clinically and statistically lower disease-specific HRQoL and greater impairment in daily activities as compared to continent patients. On average, incontinent patients scored 10 points lower on the I-QOL total score, 9 points lower on the OAB-q HRQoL score, 15 points higher on OAB-q symptom severity, and experienced 8.2 percent higher activity impairment due to their bladder condition (all p < 0.001).

**Conclusions:**

Incontinent NDO patients experience significantly lower HRQoL and activity impairment as compared to continent NDO patients.

## Background

Overactive bladder (OAB) has been defined by the International Continence Society as urgency with or without urinary incontinence (UI), usually with frequency and nocturia [1]. Compared with patients without OAB, those with OAB suffer from lower health-related quality of life (HRQoL), higher prevalence of chronic comorbidities, and substantial economic burden. Total costs were proportional to the country population size; accounting for an estimated 65.7 billion USD in the US and 4.2 billion Euros in the five largest western European countries [2,3]. In addition, OAB patients were found less likely to be employed and had significantly worse general HRQoL (EQ-5D score) as compared to patients without OAB [4,5].

Neurogenic detrusor overactivity (NDO) is defined as bladder overactivity due to a relevant neurological condition, such as multiple sclerosis (MS), spinal cord injury (SCI), Parkinson’s disease (PD), or stroke [1]. The mainstay of therapy for NDO includes the use of anticholinergic medications, along with supportive care via behavioral training, clean intermittent catheterization and the use of absorbent pads [6,7]. Other treatment options include alpha-blockers, botulinum toxin, and surgery (augmentation cystoplasty).

While the economic and HRQoL burden have been estimated in the general OAB population, the burden attributable to UI in NDO patients has not been investigated [4,8-10]. Patients with a neurogenic condition may differ in their underlying comorbidities, types of specialists accessed for care, treatment choices, and ultimately, their treatment outcomes. Thus, the objective of the current study is to assess the impact of UI on patient HRQoL, productivity, and healthcare resource use in NDO patients.

## Methods

This analysis was completed using an existing data set, the Adelphi OAB/UI Disease Specific Programmes (DSP), a cross-sectional survey, fielded between: November 2010 till February 2011. The Adelphi OAB/UI survey elicits patient-level data from the US, UK, France, Spain, and Germany, including patient demographics, physician practice trends, healthcare utilization, HRQoL, and productivity [11].

During the data collection phase, physicians were recruited via the use of publicly available lists of healthcare professionals for identification purposes, followed by a list of prespecified criteria for screening purposes (Table 1). Approximately 700 physicians completed a Patient Record Form (PRF) which provided information relating to patient demographics, employment status, bladder symptoms, disease severity, compliance, current and previous medications, and hospitalization data for the 10 to 12 most recent clinical visits for patients fulfilling the inclusion criteria over a two to three-week timeframe. Patients selected for chart abstraction and who agreed to participate completed a one-time, cross-sectional survey (i.e., the Patient Self-Completion [PSC] form) assessing OAB symptoms and their impact on daily functioning, HRQoL and work productivity. Due to the low prevalence of neurogenic bladder patients, each physician was asked to record data from at least two neurogenic patients to ensure sufficient representation in the convenience sample. Of the 7,430 charts abstracted, 4,027 (54.2%) patients completed the survey. As no patient or physician-identifiable information had been recorded within the data set (i.e., completely de-identified), no human subject approval is required.

**Table 1:** Inclusion criteria for physicians and patients to be included in the overall Adelphi OAB/UI survey*

**Physicians**	**Patients**
1. GP/ PCP; gynecologists; urologists; urogynecologists	1. Patients over 18 years old
2. 3 ≤ number of years in practice ≤ 35	2. Outpatients
3. Number of OAB/UI patients managed per week (inpatients plus outpatients)	3. Currently managed for OAB/ UI
PCP: 6	4. No females currently pregnant
Urologists/Gynecologists: 8	5. No patients suffering from OAB/UI due to urinary infection
Urologists: 2 of MS/SCI patients	
4. Seeing enough patients to be able to generate 10–12 patient record forms in 2–3 weeks	

*GP General practitioners, *PCP* Primary care physicians, *OAB* Overactive bladder, *UI* Urinary incontinence, *MS* Multiple sclerosis, *SCI* Spinal cord injury.

Patient responses to a series of validated instruments were used to evaluate HRQoL and productivity level. General HRQoL was evaluated using the EuroQoL-5D 3 level (EQ-5D 3 L) utility score [12]. EQ-5D is a standardized instrument providing a simple, generic measure of health, consisting of questions measuring five dimensions of health states (mobility, self-care, usual activities, pain/discomfort, and anxiety/depression) and a visual analog scale (VAS) measuring overall health. Utility score computation was derived from responses to all questions excluding the VAS. Disease-specific HRQoL was elicited using the Incontinence Quality of Life questionnaire (I-QOL) total score and the Overactive Bladder Questionnaire (OAB-q) symptom severity score and HRQoL score [13,14]. I-QOL and OAB-q are 22-item and 33-item standardized instruments assessing the effect of urinary incontinence and symptom bother/HRQoL impact of OAB on patients’ life, respectively. Finally, productivity was assessed using the Work Productivity and Activity Impairment (WPAI) questionnaire [15]. WPAI evaluates percent daily activity impairment, percent impairment while working (i.e., presenteeism), percent work time missed (i.e., absenteeism), and percent overall work impairment (i.e., combination of absenteeism and presenteeism). The WPAI questionnaire was adapted to OAB/UI patients by specifically reflecting the impact of OAB/ UI on work productivity and daily activity (e.g., “During the past seven days, how many hours did you miss from work because of your bladder symptoms?”). Published scoring algorithms were used to generate total and domain scores for each measure [16-19]. The US algorithm was used to compute utility scores for the analytic sample because: 1) the possible range of the mapped score is a balance between using the UK algorithm and other utility deriving instruments (e.g., Health Utility Index II, III, and the Short Form-36), and 2) utility scores could be consistently estimated across international patients. Table 2 lists the possible score ranges and interpretation for each measure.

**Table 2:** **Instrument score range and interpretation**^**a,b**^

**Outcome measures**		**Score range**	**Interpretation**
General Health Utility	EQ-5D 3 L utility score^c^	−0.11 – 1	General health ↑ as score ↑
Disease-specific QOL	I-QOL total score	0 – 100	HRQoL ↑ as score ↑
	OAB-q symptom severity score	0 – 100	Symptom severity ↓ as score ↑
	OAB-q HRQoL score	0 – 100	HRQoL ↑ as score ↑
Productivity	% activity impairment due to their bladder condition	0 – 100	Productivity ↓ as score ↑
	% work time missed due to their bladder condition	0 – 100	Productivity ↓ as score ↑
	% impairment while working due to their bladder condition	0 – 100	Productivity ↓ as score ↑
	% overall work impairment due to their bladder condition	0 – 100	Productivity ↓ as score ↑

^a^*QOL*, Quality of life; *EQ-5D*, EuroQoL-5D; *I-QOL*, Incontinence Quality of Life questionnaire; *OAB-q*, Overactive Bladder questionnaire; *HRQoL*, Health-related quality of life.

^b^↓ indicates decrease, ↑ indicates increase.

^c^Derived from the 5 questions representing 5 dimensions of health states in the EQ-5D instrument using standardized scoring algorithm.

Health resource utilization was evaluated using the following measures: 1) proportion of patients that ever had OAB-related surgery; 2) proportion of patients having OAB-related hospitalization over the past 12 months; 3) proportion of patients currently using incontinence pads (at the time of completing the survey); 4) number of pads used per week; 5) proportion of patients that had switched anticholinergics used for OAB due to inadequate response or adverse drug reactions; and 6) number of OAB-related physician visits over the past 3 months. Hospitalizations, surgery, and anticholinergic history were derived from chart review, while the remainder of items was patient-reported.

### Patient selection

To be included in the specific analytic sample for this research, patient data had to: (1) include an OAB diagnosis with or without incontinence associated with an underlying neurological condition; (2) have completed the PSC form; and (3) have a history of anticholinergics use for OAB. There has been limited focus on HRQoL and healthcare resource use in OAB patients exposed to more treatment options (i.e., whose disease condition has not been appropriately controlled with anticholinergics). Hence, we targeted this specific population to provide public data for researchers to use in future studies. Data from patients with functional, flow, stress, or mixed-type incontinence were excluded. Incontinence status (yes/no) was determined from a question on the PSC form, “Are there times when you experience leakages, however small the amount?” The frequency of incontinent episodes was obtained from the question, “Thinking about the last week…On average, what is the total number of leakages you experienced over a 24 hour period?” Only those patients reporting leakages were eligible to report the number of episodes in the second question.

### Statistical analysis

First, Chi-square tests or Fisher’s exact tests were used for categorical variables, while Student’s t-tests or Wilcoxon rank-sum tests were used for continuous variables to explore differences between continent and incontinent patients in terms of HRQoL, productivity measures, and healthcare resource utilization to assess the relationship between incontinence and these factors. To further control for confounders that may potentially bias the relationship between incontinent status and health outcomes, multivariate ordinary least square (OLS) regression models were employed to evaluate associations between number of incontinent episodes and quality of life/productivity loss related to OAB/UI. Other secondary independent variables (including time since diagnosis, age, sex, race, education, insurance status, and comorbidities) used in the regression analyses were selected based on: 1) univariate regression analyses and descriptive statistics; 2) theoretically sound constructs possibly predictive of the endpoints; and 3) variables available in the survey.

The magnitude of score changes reported in the literature were used to assess whether effect sizes reached or exceeded the minimal clinically important differences (MCID) for each measure: EQ-5D utility score—0.03 to 0.07 [20,21]; I-QOL subscales—4 to 11 [13]; OAB-q HRQoL score—5 [22,23]; OAB-q symptom severity score—10 [22,23]. MCIDs for WPAI have been validated only in patients with insomnia and Crohn’s disease [24,25], and thus were deemed insufficiently reliable to be used for comparisons.

To test the robustness of these associations, sensitivity analyses were conducted on the incontinence variable by categorizing it as ordinal (continent; incontinent with 0 to 4 daily episodes; incontinent with more than 4 daily episodes) and continuous (number of UI episodes per week). Additional sensitivity analyses were also conducted using negative binomial models in lieu of OLS regression models. To minimize sample size loss due to missing data, median (for number of pads used and number of daily incontinent episodes) and multiple imputations (for all other study variables with missing data) were employed to predict values for missing data with respect to variables used in the regression and sensitivity analyses. Median imputation was used for the two variables because their values were conditional on whether they had used any pads/had any incontinent episodes; missing data from both of the dichotomous variables were replaced using multiple imputation. SAS version 9.2 (Cary, NC) and STATA version 12.1 (College Station, TX) were used for data management and analysis, respectively. Significance level was set at p < 0.05.

## Results

**Figure 1:**
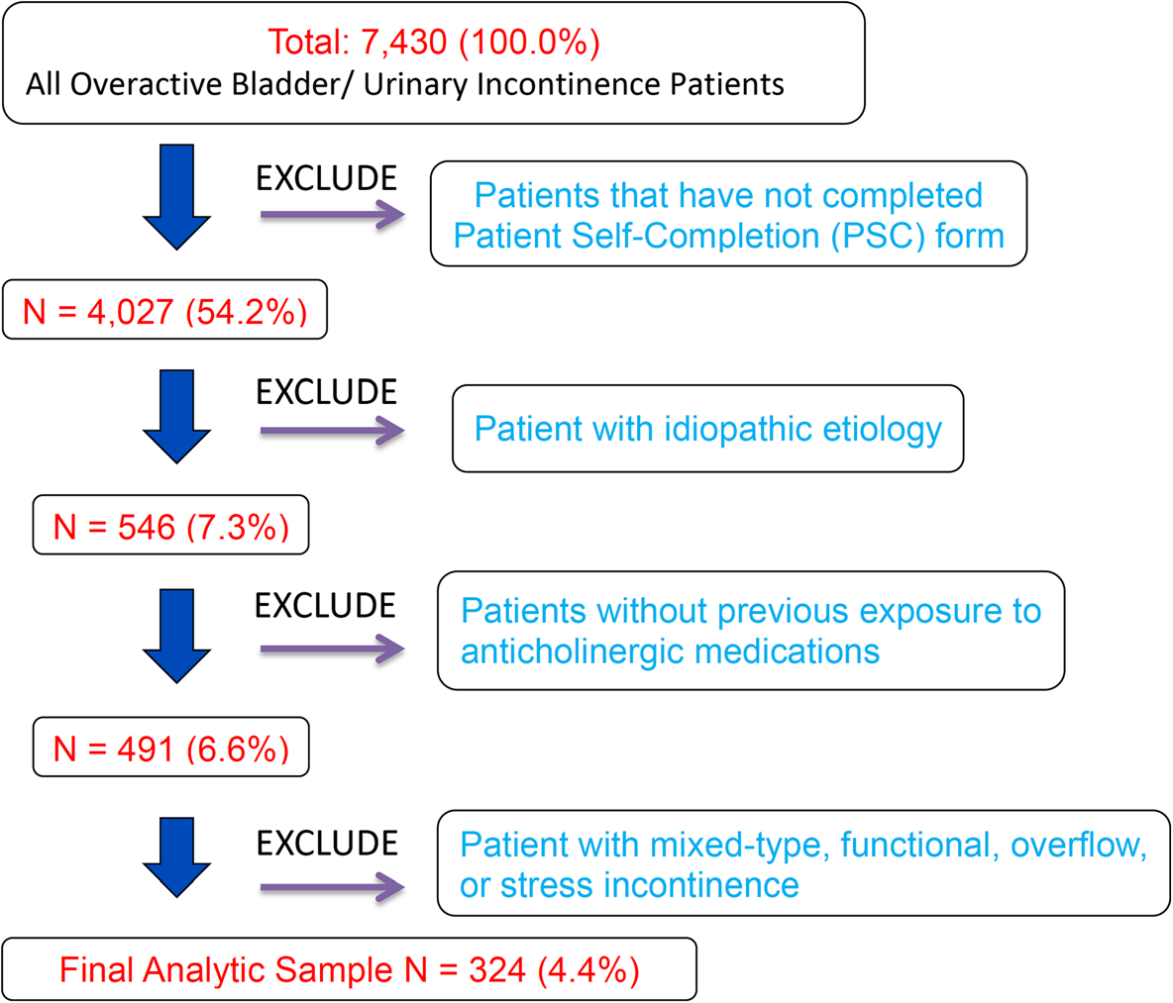
Patient cohort selection flowchart.

The final analytic sample included data from 324 patients (54.1% of 599 patients completed the PSC form) (Figure 1). Compared with PSC responders, PSC non-responders were on average older (56.1 vs. 53.0, p = 0.02); consisted of more White/Caucasian (83.1% vs. 80.9%), minority races (7.0% vs. 2.8%), and less Hispanic/Latino (1.1% vs. 8.0%) (p_ethnicity_ < 0.001); had more patients with a underlying neurological diagnosis of PD (15.6% vs. 9.3%) and stroke (13.1% vs. 9.6%), and less patients with MS (42.2% vs. 52.8%) and SCI (16.4% vs. 21.3%) (p_neuro_ = 0.003). However, the effect size of these differences was not clinically meaningful. Time since diagnosis was comparable (p = 0.24) between responders and non-responders. Patients from the US and Western European countries accounted for 27.5% (n = 89) and 72.5% (n = 235) of the final sample, respectively. The study sample averaged 53.5 years of age and consisted of 56.2% female, 80.9% non-Hispanic whites, and 75.1% incontinent patients. A primary neurological diagnosis of PD, MS, SCI, or stroke was identified in 92.9% of the patients, with MS patients comprising over half of the sample (Table 3).

**Table 3:** **Distribution of underlying neurological conditions among continent and incontinent NDO patients**^**a-c**^

**Neurological conditions**	**Continent**	**Incontinent**
	**(n (% all continent patients))**	**(n (% all incontinent patients))**
Parkinson’s Disease (PD)	6 (7.7%)	23 (9.8%)
Multiple Sclerosis (MS)	43 (55.1%)	122 (51.9%)
Spinal Cord Injury (SCI)	14 (17.9%)	52 (22.1%)
Stroke	10 (12.8%)	21 (8.9%)
Other	4 (5.1%)	12 (5.1%)
Unknown	1 (1.3%)	5 (2.1%)
Total	78 (100.0%)	235 (100.0%)

^a^301 (92.9%) out of 324 NDO patients had a primary neurological condition of either PD, MS, SCI or stroke.

^b^11 patients out of the 324 patients had unknown incontinence status.

^c^Among all SCI patients, 29% had complete SCI, 61% had incomplete SCI, while 10% had unknown injury status.

No statistically significant differences in baseline demographics were noted between continent and incontinent patients (Table 4). Bivariate analyses showed that disease-specific quality of life was significantly lower and that daily activity impairment was significantly higher in incontinent patients compared with continent patients (Table 5). Specifically, incontinent OAB patients had an average I-QOL total score of 52, compared with 63 in continent patients (p < 0.001), In addition, incontinent patients had a significantly higher OAB-q symptom severity score and a lower OAB-q HRQoL score as compared to continent patients. Activity impairment, as assessed by the WPAI, was significantly higher in incontinent as compared with continent patients (52 vs 44 points, p < 0.05). Finally, in the US sample, the EQ-5D utility score was significantly lower in incontinent as compared to continent patients (0.68 vs. 0.82, p < 0.001). All of these differences were clinically significant (except for WPAI outcomes in which the MCIDs were relatively unclear), as defined by the published MCID of the respective instruments.

**Table 4:** **Patient demographics by incontinence status**^**a**^

**Variables**			**Incontinence status**^**b**^	
			**Continent**	**Incontinent**
			**(n = 78)**	**(n = 235)**
**Age (mean (sd))**	USA		55.8 (17.4)	54.9 (15.9)
	Europe		49.9 (16.5)	54.1 (16.2)
	Total		51.5 (16.9)	54.3 (16.1)
**Male (n (%))**	USA		11 (52.4%)	23 (35.4%)
	Europe		24 (42.1%)	79 (47.0%)
	Total		35 (44.9%)	102 (43.8%)
**Diagnosed ≥ 1 year ago**	USA**		9 (52.9%)	55 (88.7%)
**(n (%))**	Europe		38 (73.1%)	110 (68.8%)
	Total		47 (68.1%)	165 (74.3%)
**Ethnicity (n (%))**	USA	White/Caucasian	11 (52.4%)	45 (69.2%)
		Hispanic/Latino	4 (19.0%)	5 (7.7%)
		Afro/Caribbean	5 (23.8%)	13 (20.0%)
		Others	1 (4.8%)	2 (3.1%)
	Europe	White/Caucasian	48 (84.2%)	148 (87.1%)
		Hispanic/Latino	7 (12.3%)	9 (5.3%)
		Afro/Caribbean	2 (3.5%)	7 (4.1%)
		Others	0 (0.0%)	6 (3.5%)
	Total	White/Caucasian	59 (75.6%)	193 (82.1%)
		Hispanic/Latino	11 (14.1%)	14 (6.0%)
		Afro/Caribbean	7 (9.0%)	20 (8.5%)
		Others	1 (1.3%)	8 (3.4%)
**Education (n (%))**	USA	Less than high school	1 (5.0%)	1 (1.8%)
		High school	6 (30.0%)	22 (39.3%)
		College or more	13 (65.0%)	33 (58.9%)
	Europe	Less than high school	6 (11.1%)	25 (15.5%)
		High school	18 (33.3%)	65 (40.4%)
		College or more	30 (55.6%)	71 (44.1%)
	Total	Less than high school	7 (9.5%)	26 (12.0%)
		High school	24 (32.4%)	87 (40.1%)
		College or more	43 (58.1%)	104 (47.9%)
**Have any insurance (n (%))**	USA		18 (100.0%)	55 (98.2%)
	Europe		43 (76.8%)	125 (76.7%)
	Total		61 (82.4%)	180 (82.2%)

^a^Bivariate analyses comparing distribution of demographic variables between incontinent versus continent group.

^b^11 out of 324 NDO patients had unknown incontinent status.

*p < 0.05 **p < 0.01 ***p < 0.001 in chi-square test, Fisher’s exact test, t-test, or Wilcoxon rank-sum test as appropriate.

**Table 5:** **Relationship between Quality of Life (QOL) and productivity measures and sncontinence status**^**a**^

**Variables**			**Incontinence status**^**b**^	
			**Continent**	**Incontinent**
			**(n = 78)**	**(n = 235)**
**General QOL**	EQ-5D utility score‡‡	USA*	0.82 (0.22)	0.68 (0.24)
		Europe	0.73 (0.22)	0.70 (0.22)
		Total	0.75 (0.22)	0.70 (0.22)
**Disease-specific QOL**	I-QOL score‡‡	USA**	72 (19)	57 (23)
		Europe***	59 (17)	50 (19)
		Total***	63 (19)	52 (20)
	OAB-q: Symptom severity‡‡	USA*	37 (19)	49 (19)
		Europe***	41 (20)	56 (18)
		Total***	40 (20)	54 (19)
	OAB-q: HRQL total‡‡	USA***	79 (15)	62 (23)
		Europe	63 (19)	57 (19)
		Total**	67 (20)	58 (20)
**Productivity**	WPAI activity impairment‡‡	USA	34 (26)	46 (28)
		Europe	48 (22)	55 (25)
		Total*	44 (24)	52 (26)
	WPAI overall impairment	USA	53 (32)	32 (19)
	(among patients on paid employment: n (continent) = 19, n(incontinent) = 45)‡‡	Europe	49 (22)	50 (28)
		Total	50 (23)	43 (26)
	WPAI impairment while working (among patients on paid employment: n (continent) = 19, n(incontinent) = 45)‡‡	USA	38 (33)	33 (18)
		Europe	39 (22)	46 (24)
		Total	39 (24)	42 (23)
	WPAI work time miss (among patients on paid employment: n (continent) = 19, n(incontinent) = 45)‡‡	USA	15 (26)	1 (2)
		Europe	8 (13)	12 (21)
		Total	10 (15)	8 (18)
	Proportion of patients on paid employment‡∆	USA	5 (23.8%)	15 (24.2%)
		Europe	14 (24.6%)	30 (17.9%)
		Total	19 (24.4%)	45 (19.6%)

^a^Bivariate analyses comparing burden of illness between incontinent versus non-incontinent group.

^b^11 out of 324 NDO patients had unknown incontinent status.

*p < 0.05 **p < 0.01 ***p < 0.001 in chi-square test, Fisher’s exact test, t-test, or Wilcoxon rank-sum test as appropriate.

‡Data are presented as number (percentage).

‡‡Data are presented as mean (standard deviation).

∆Retired patients (n = 1) who reported “under paid employment” were analyzed as paid employees.

Slightly over 10% of the patients in the sample reported an OAB-related hospitalization in the past year, although there was no statistically significant difference in reported hospitalizations between continent and incontinent patients (Table 6). Similar rates were reported for OAB-related surgeries. On average, patients report 2 OAB-related office visits per year, with no statistically significant different between those who were continent compared to those who were incontinent. As expected, incontinence pad use was significantly higher among incontinent patients. On average, incontinent patients used 9.7 pads per week, compared with 1.8 pads per week for continent patients (p < 0.001). Reported hospitalization rates were higher for US compared to European patients.

**Table 6:** **Relationship between health resource utilization and incontinence status**^**a**^

**Variables**		**Incontinence status**^**b**^	
		**Continent**	**Incontinent**
		**(n = 78)**	**(n = 235)**
**Proportion of patients having OAB-related hospitalization experience during the past 12 months‡**	USA	1 (5.0%)	3 (4.6%)
	Europe	9 (15.8%)	31 (18.2%)
	Total	10 (12.8%)	34 (14.5%)
**Proportion of patients that ever had OAB-related surgery‡**	USA	2 (9.5%)	10 (15.9%)
	Europe	6 (10.5%)	11 (6.5%)
	Total	8 (10.3%)	21 (9.1%)
**Proportion of patients that had switched anticholinergics used for OAB due to efficacy/side effects‡**	USA	6 (28.6%)	15 (23.1%)
	Europe	18 (31.6%)	46 (27.1%)
	Total	24 (30.8%)	61 (26.0%)
**Proportion of patients currently using pads (at the time of completing the survey) ‡**	USA**	4 (19.0%)	40 (61.5%)
	Europe***	6 (10.5%)	127 (75.6%)
	Total***	10 (12.8%)	167 (71.7%)
**Number of OAB-related physician visits over the past 3 months‡‡**	USA	1.3 (1.2)	1.5 (1.3)
	Europe	2.2 (2.4)	2.5 (2.3)
	Total	2.0 (2.1)	2.2 (2.0)
**Number of pads used per week‡‡**	USA	4.5 (12.2)	10.8 (14.4)
	Europe***	0.8 (2.4)	9.3 (9.0)
	Total***	1.8 (6.7)	9.7 (10.8)

^a^Bivariate analyses comparing burden of illness between incontinent versus non-incontinent group.

^b^11 out of 324 NDO patients had unknown incontinent status.

*p < 0.05 **p < 0.01 ***p < 0.001 in chi-square test, Fisher’s exact test, t-test, or Wilcoxon rank-sum test as appropriate.

‡Data are presented as number (percentage).

‡‡Data are presented as mean (standard deviation).

After adjustment for age, sex, ethnicity, education level, insurance status, disease duration, country, and comorbidities, incontinence status remained significantly associated with lower disease-specific QOL and higher impairment in daily activities (Table 7). Compared with continent patients, incontinent patients on average scored 10 points lower on the I-QOL total score, 9 points lower on the OAB-q HRQoL score, 15 points higher on OAB-q symptom severity, and experienced 8.2% higher activity impairment due to their bladder condition (all p < 0.05). These differences were all clinically meaningful based on previously published MCIDs [13,20-23]. Sensitivity analyses confirmed these statistically significant and clinically meaningful relationships (Tables 7 and 8). Compared with continent patients, patients with more than 4 daily UI episodes had on average 14 points lower I-QOL total score, 12 points lower OAB-q HRQoL score, 20 points higher OAB-q symptom severity, and 14% higher activity impairment related to their bladder symptoms (all p < 0.001) (Table 7). Although general HRQoL was not statistically significantly different between incontinent and continent patients, patients with more than 4 daily UI episodes (compared with patients with no leakages) on average had 0.07 lower EQ-5D utility score (p < 0.05) (Table 7). Additionally, number of UI episodes was statistically significantly associated with lower EQ-5D utility scores: one additional episode was associated with a 0.01 point decrease in utility (p < 0.05). While qualitative differences were observed in work productivity impairment, these differences were not statistically significant.

**Table 7:** **Primary analysis: adjusted associations between incontinence status and quality of life (QOL)/productivity measures using linear regression models (n = 324)**^**†**^

**Quality of life outcomes**	**Incontinence status**						
	**Base-case analysis**		**Sensitivity analysis**				
	**Incontinence (Yes vs. No)**	**R**^**2**^	**Average number of UI episodes per day**	**R**^**2**^	**Number of UI episodes (Ordinal)**^**c**^		**R**^**2**^
**EQ-5D utility score**^**a**^	−0.04 (0.03)	0.21	−0.01 (0.00)*	0.22	0 ≤ n ≤ 4	−0.02 (0.03)	0.22
					n > 4	−0.07 (0.03)*	
**I-QOL score**^**a**^	−10.39 (2.52)***	0.25	−1.03 (0.29)***	0.24	0 ≤ n ≤ 4	−8.47 (2.69)**	0.26
					n > 4	−13.66 (2.97)***	
**OAB-q symptom severity score**^**a**^	15.01 (2.58)***	0.22	1.65 (0.29)***	0.22	0 ≤ n ≤ 4	11.92 (2.72)***	0.25
					n > 4	20.27 (3.01)***	
**OAB-q HRQL total score**^**a**^	−8.69 (2.72)**	0.19	−1.03 (0.30)***	0.19	0 ≤ n ≤ 4	−6.63 (2.89)*	0.20
					n > 4	−12.22 (3.18)***	
**WPAI – percent activity impairment due to problem**^**a**^	8.23 (3.41)*	0.15	1.40 (0.38)***	0.17	0 ≤ n ≤ 4	4.88 (3.62)	0.17
					n > 4	13.95 (4.03)**	
**WPAI – percent work time miss due to problem‡**^**a,b**^	3.69 (5.66)	0.22	1.51 (1.15)	0.24	0 ≤ n ≤ 4	3.51 (5.90)	0.22
					n > 4	4.71 (8.98)	
**WPAI – percent impairment while working due to problem‡**^**a,b**^	3.70 (7.13)	0.28	3.14 (1.42)*	0.34	0 ≤ n ≤ 4	1.69 (7.16)	0.31
					n > 4	15.93 (11.33)	
**WPAI – percent overall work impairment due to problem‡**^**a,b**^	3.04 (8.96)	0.27	1.94 (1.79)	0.28	0 ≤ n ≤ 4	3.30 (9.30)	0.27
					n > 4	1.21 (13.88)	

†All analyses adjusted for time since diagnosis, age, male sex, race, education, insurance status, country, and whether having the following comorbidities: psychological/psychiatric conditions, hypertension, diabetes, and dementia.

‡Comorbidities were not included as covariates.

^a^Mean regression coefficient (non-exponentiated) (standard error).

^b^Population restricted to patients that were on paid employment (n = 68).

^c^Reference group: patients that reported no leakages.

*p < 0.05 **p < 0.01 ***p < 0.001.

**Table 8:** **Sensitivity analysis: adjusted associations between incontinence status and quality of life (QOL)/ productivity measures using negative binomial regression models (n = 324)**^**†**^

**Quality of life outcomes**	**Incontinence status**			
	**Base-case analysis**	**Sensitivity analysis**		
	**Incontinence (Yes vs. No)**	**Average number of UI episodes per day**	**Number of UI episodes (Ordinal)**^**c**^	
**EQ-5D utility score**^**a**^	0.95 (0.15)	0.99 (0.02)	0 ≤ n ≤ 4	0.97 (0.16)
			n > 4	0.91 (0.17)
**I-QOL score**^**a**^	0.83 (0.04)***	0.98 (0.01)**	0 ≤ n ≤ 4	0.86 (0.05)**
			n > 4	0.78 (0.05)***
**OAB-q symptom severity score**^**a**^	1.38 (0.08)***	1.03 (0.01)***	0 ≤ n ≤ 4	1.30 (0.08)***
			n > 4	1.52 (0.10)***
**OAB-q HRQL total score**^**a**^	0.87 (0.05)**	0.98 (0.01)**	0 ≤ n ≤ 4	0.90 (0.05)
			n > 4	0.81 (0.05)**
**WPAI – percent activity impairment due to problem**^**a**^	1.21 (0.10)*	1.03 (0.01)**	0 ≤ n ≤ 4	1.12 (0.10)
			n > 4	1.34 (0.13)**
**WPAI – percent work time miss due to problem‡**^**a,b**^	2.77 (3.11)	1.46 (0.48)	0 ≤ n ≤ 4	2.89 (3.29)
			n > 4	1.87 (3.86)
**WPAI – percent impairment while working due to problem‡**^**a,b**^	1.04 (0.26)	1.08 (0.05)	0 ≤ n ≤ 4	0.99 (0.25)
			n > 4	1.39 (0.52)
**WPAI – percent overall work impairment due to problem‡**^**a,b**^	1.03 (0.39)	1.02 (0.07)	0 ≤ n ≤ 4	1.06 (0.40)
			n > 4	0.81 (0.45)

†All analyses adjusted for time since diagnosis, age, male sex, race, education, insurance status, country, and whether having the following comorbidities: psychological/psychiatric conditions, hypertension, diabetes, and dementia.

‡Comorbidities were not included as covariates.

^a^Incidence rate ratio (standard error).

^b^Population restricted to patients that were on paid employment (n = 68).

^c^Reference group: patients that reported no leakages.

*p < 0.05 **p < 0.01 ***p < 0.001.

## Discussion

In the current study, incontinent patients with an underlying neurological condition were found to have significantly lower disease-specific HRQoL and greater activity impairment compared with continent patients. However, the differences in general HRQoL and work productivity were not statistically significant. One potential explanation is that the severity of the patients’ underlying neurological disease may limit the ability to distinguish the incremental impact of their urinary incontinence. Secondly, it is possible that the EQ-5D may lack the sensitivity to detect such differences. Thirdly, the study was not specifically powered to observe such differences, particularly in the case of the WPAI. All analyses on absenteeism, presenteeism, and overall work impairment included only self-identified paid employees (n = 66). In general, patients with NDO may have a lower employment rate due to their underlying neurological conditions. In this analysis, the proportion of NDO patients on paid employment was significantly lower as compared to other OAB patients (21% vs. 36%, p < 0.001). Further investigations to verify these associations are recommended on a larger sample of NDO patients.

The current study is, to our knowledge, the first study that systematically evaluates the HRQoL, productivity burden, and healthcare resource utilization between incontinent and continent NDO patients. Previous investigations focused on estimating the burden of illness in the general OAB population and identified significant relationships between incontinence and both general and disease-specific quality of life. Specifically, Coyne et al. found that both men and women with incontinent OAB were significantly more likely to report OAB symptom-specific bother (odds ratio (OR) = 1.81 in men and 1.92 in women, respectively) [10]. In another investigation by Coyne and colleagues, significantly lower utility score and greater bother in all subscales of OAB-q was detected in incontinent OAB patients as compared to continent OAB patients [9]. By estimating SF-36 domain scores, Stewart et al. found clinically significant differences in 7 out of 8 domain scores between incontinent and continent patients [8]. In the current study, general quality of life as assessed by the EQ-5D did not significantly differ in the NDO patients. As previously mentioned, the total burden of illness in neurogenic patients includes the neurologic and urinary condition, along with any additional comorbidities. Thus, the impact of urinary incontinence itself may be difficult to ascertain among these patients. Furthermore, the sensitivity of the EQ-5D 3 L may be limited to assess such differences, in contrast to the disease-specific instruments. The sensitivity of alternative utility instruments such as the OAB-5D, developed specifically for an overactive bladder population from the OAB-q, may be worth exploring in future studies [26]. In addition, as the current study focuses on the incremental impact of urinary incontinence (as opposed to frequency and other symptoms of OAB), a utility measure developed from an incontinence-specific instrument such as the I-QOL may be another viable alternative.

Major strengths of this study included the direct specification of patient diagnoses from their physicians, the strict inclusion criteria applied during patient recruitment, and the exclusion of mixed-type incontinent patients. Moreover, the effect of recall bias on the frequency of incontinence episodes was relatively low, as it was determined using a recall period of one week as compared to four weeks in previous investigations.

The current study was also subjected to limitations. The survey data were collected using non-random sampling methods, and the strict inclusion criteria for the subsequent analysis may limit the generalizability of results to those with similar characteristics as the study population. Secondly, causal relationships between incontinence status and quality of life cannot be verified due to the cross-sectional nature of the survey data. Thirdly, the MCID value pertaining to EQ-5D 3 L was based on chronic disease patients other than OAB patients. Ideally, future research might validate the instrument in the OAB population, particularly among patients with a neurological condition. Finally, statistical correlations among patients recruited by the same physicians may potentially influence the study results.

## Conclusion

The current investigation found that in NDO patients in Western Europe and the US, urinary incontinence was associated with clinically and statistically lower disease-specific QOL and greater daily activity impairment. These associations were robust as demonstrated by the sensitivity analyses. These results suggest that—in addition to the patients’ underlying neurological conditions, their urinary incontinence contributes to a significant degree of humanistic burden. Healthcare providers may consider such impacts on the patients’ quality of life when selecting appropriate treatments.

## Abbreviations

NDO: Neurogenic detrusor overactivity; UI: Urinary incontinence; HRQoL: Health-related quality of life; HRU: Health resource use; OAB: Overactive bladder; EQ-5D: EuroQoL-5D; I-QOL: Incontinence Quality of Life questionnaire; OAB-q: Overactive Bladder Questionnaire; WPAI: Work Productivity and Activity Impairment; OLS: Ordinary least squares; MCID: Minimal clinically important differences; SD: Standard deviation; MS: Multiple sclerosis; SCI: Spinal cord injury; PD: Parkinson’s disease; DSP: Disease-Specific Programmes; PRF: Patient record form; PSC: Patient self-completion; EQ-5D 3 L: EuroQoL-5D 3 level; VAS: Visual analog scale; OR: Odds ratio.

## Competing interests

Throughout the study completion process, Derek H Tang, MS, PhD Candidate, served as a paid intern for the department of Global Health Outcomes Strategy and Research, Allergan Inc.; Danielle Colayco, PharmD, MS, served as a full-time employee, followed by transition into a paid consultant for the department of Global Health Outcomes Strategy and Research, Allergan Inc.; Denise Globe, PhD, and Vaishali Patel, PharmD, MS, have been full-time employees of the department of Global Health Outcomes Strategy and Research, Allergan Inc.; Michael B Chancellor, MD, served as a consultant and investigator with Allergan Inc. The descriptions above refer specifically to the approaches and analysis for this specific manuscript to address the research questions of quality of life and utilization between those with and without incontinence; the actual broader study (i.e., the process of creating the Adelphi OAB/UI data set) was developed and fielded by Adelphi Values with input from Vaishali Patel and Tina Chiang from Allergan Inc.

## Authors’ contribution

DHT made substantial contributions to the study design, analysis, interpretation of data, and drafting the manuscript; DC, DG, and VP made substantial contributions to the study design; DC, DG, VP, JP, and MBC made critical revision of the manuscript for important intellectual content. All authors have read and approved the final version of the manuscript.

## Authors’ information

At the time of the analysis for this paper, Danielle Colayco was an employee of Allergan, Inc.

## Pre-publication history

The pre-publication history for this paper can be accessed here:

http://www.biomedcentral.com/1471-2377/14/74/prepub
